# Extraction, Structural Characterization, and Immunomodulatory Activity of a High Molecular Weight Polysaccharide From *Ganoderma lucidum*

**DOI:** 10.3389/fnut.2022.846080

**Published:** 2022-03-25

**Authors:** Guo Liu, Jun Zhang, Qixin Kan, Mingyue Song, Tao Hou, Siyu An, Hongyu Lin, Hongzhang Chen, Liuyun Hu, Jie Xiao, Yunjiao Chen, Yong Cao

**Affiliations:** ^1^Guangdong Provincial Key Laboratory of Nutraceuticals and Functional Foods, College of Food Science, South China Agricultural University, Guangzhou, China; ^2^College of Horticulture, South China Agricultural University, Guangzhou, China; ^3^Infinitus China Co., Ltd., Guangzhou, China

**Keywords:** *Ganoderma lucidum*, high molecular weight polysaccharides, purification, structure, immunomodulatory activity

## Abstract

*Ganoderma lucidum* polysaccharides (GLP) exhibited excellent immunomodulatory activity. Unfortunately, the structure and immunomodulatory activity of GLP are still unclear. GLP was separated into two fractions [high Mw Restriction Fragment Length Polymorphism (RGLP) and low Mw EGLP] using 10 kDa cut-off ultrafiltration membrane. Although the RGLP content was low in GLP, the immunomodulatory activity in RGLP was significantly higher than that of EGLP. Moreover, RGLP was further separated *via* the Sephacryl column to obtain RGLP-1 showed the best immunomodulatory activity in the macrophage RAW264.7 model. Structural analysis revealed that RGLP-1 was 3,978 kDa and mainly consisted of glucose. Periodate oxidation, Smith degradation, and methylation results indicated that RGLP-1 is a β-pyran polysaccharide mainly with 1→3, 1→4, 1→6, and 1→3, 6 glycosyl bonds at a molar ratio of 40.08: 8.11: 5.62: 17.81. Scanning electron microscopy, atomic force microscopy, and Congo red experiments revealed that RGLP-1 intertwined with each other to form circular aggregates and might possess a globular structure with triple-helix conformation in water. Overall, these results provide RGLP-1 as a potential functional food ingredient or pharmaceutical for immunomodulatory.

## Introduction

*Ganoderma lucidum* (*G. lucidum*) is both food and traditional Chinese medicine (1). It has a long history of being used as soup, tea, and liquor food raw material in Shandong, Anhui, and other regions of China. There are a large number of commercially available functional foods with *G. lucidum* as raw materials in Asian counties (2, 3). Until now, the number of functional foods products containing *G. lucidum* has reached about 1,060 according to the published data from Chinese Administration for Market Regulation. The annual sale of *G. lucidum* products exceeded $2.5 billion in 2013 (5). In recent decades, *G. lucidum* exhibits valuable biological activities, such as immunomodulatory, antitumor, antioxidant, antiaging, anti-inflammatory, and hypoglycemic effects (6, 7). In modern pharmacological and functional food investigations, a number of bioactive compounds were separated from *G. lucidum*, including polysaccharides, triterpenoids, protein, sterols, nucleosides, and alkaloids (8–10). Polysaccharides stand for one of the most abundant components in *G. lucidum*, contributing to a major active ingredient with numerous healthy benefits, particularly for the prevention and treatment of immunomodulation (11) and antitumor properties (12, 13). It has been reported that the biological activity of *G. lucidum* polysaccharides (GLP) relies on their chemical composition (14).

Currently, there is no general agreement on the relationships between the structural features and biological activities of GLP (2). The structure of polysaccharides is complex and diverse, which is determined by molecular weight, monosaccharide composition, glycoside bond structure, configurations of main and branch chains, and branch type (15). Expectedly, the bioactivity depended on molecular size and chemical structures (16). A study reported that a heteroglycans (PL-1) with a molecular weight of 8.3 kDa isolated from fruiting bodies of *G. lucidum* had an immune-stimulating activity in mice (17). Researchers purified a homogeneous polysaccharide with an average molecular weight of 44.4 kDa from *G. lucidum* fruiting bodies and found that this polysaccharide exhibited excellent antitumor and immunomodulatory activities using a mouse model (18). A homogeneous *Ganoderma atrum* polysaccharide (PSG-1) with an average molecular weight of 1,013 kDa has strong immunomodulatory, antitumor, and antioxidant activities (1, 19, 20). A water-soluble polysaccharide (GSP-6B) with a molecular mass of 1,860 kDa was isolated from the fruiting bodies of *Ganoderma sinense*, an *in vitro* immunomodulating activity assay revealed that GSP-6B could significantly induce the release of IL-1β and TNF-α in human peripheral blood mononuclear cells in a dose-dependent manner, suggesting that GSP-6B had the potential as adjuvant therapy medicines against tumors (21). To sum up, it has been reported that both high and low *Ganoderma* polysaccharides have immunomodulatory activity. A previous study showed that high molecular weight was probably not necessary to obtain immunoregulatory activities (22). On the contrary, a recent study has shown that bioactivities of polysaccharides are closely correlated to their molecular weight, the larger the molecular weight of GLP, the higher the bioactivity function (2). To date, this query that what molecular weight of polysaccharide has the best immunomodulatory activity has been controversial, the relationship between the molecular weight of GLP and immunobiological activity is still unclear.

In this work, GLP was obtained using a novel continuous phase transition extraction technology. We then used ultrafiltration purification and RAW264.7 macrophages model *in vitro* to investigate the immunobiological activity. Fraction Restriction Fragment Length Polymorphism (RGLP) with the greatest immunobiological activity was further purified by Sephacryl S-500 HR column chromatography. The structure characterization of sub-fraction RGLP-1 was investigated systematically by high-performance gel permeation chromatography (HPGPC), gas chromatography–mass spectroscopy (GC–MS), methylation analysis, Fourier transform-infrared spectroscopy (FT-IR), Congo red test, circular dichroism (CD), scanning electron microscopy (SEM), and atomic force microscopy (AFM). Therefore, these should all help further clarify the structure-effective relationship of GLP. Further, RGLP-1 was potential commercial functional food ingredient for immunoregulation.

## Materials and Methods

### Materials and Chemicals

The fruiting bodies of *G. lucidum* were supplied by Infinitus China Co., Ltd. (Guangzhou, China) and cut into small pieces (sieved through 3 mesh screens). The *G. lucidum* pieces were dried at 60^°^C in the oven and stored at room temperature until analysis. A multifunctional continuous phase transition extraction apparatus was supplied by Guangdong Provincial Key Laboratory of Nutraceuticals and Functional Foods.

Cut-off ultrafiltration membrane (10 kDa, Vivaflow 50) and Sephacryl S-500 HR were purchased from Sartorius Life Sciences (Goettingen, Sartorius) and GE Healthcare Life Sciences (Uppsala, Sweden), respectively. Macrophage RAW264.7 cells line was purchased from the Chinese Academy of Sciences Cell Bank. Dextrans with different molecular weights (5, 25, 50, 80, 150, 270, 410, 670, and 1,800 kDa) and the monosaccharide standards (xylose, fructose, galactose, arabinose, mannose, and glucose) were acquired from Sigma-Aldrich (MO, United States). Dulbecco’s modified Eagle’s minimum (DMEM), penicillin, 3-(4, 5-dimethylthiazol-2-yl)-2, 5-diphenyltetrazolium bromide (MTT), streptomycin, fetal bovine serum (FBS), and dimethyl sulfoxide (DMSO) were obtained from Gibco (Carlsbad, California, United States). All of the other reagents were analytical grade.

### Extraction and Separation of Polysaccharides

The extraction of crude GLP was performed using a continuous phase transition extraction apparatus on a pilot scale. Air-dried fruiting bodies of *G. lucidum* pieces (2.0 kg) were placed in a 3 L extraction kettle. The extraction conditions were as follows: solvent, distilled water; temperature, 100^°^C; time, 4 h; flow rate, 28 L/h. The aqueous extracts were concentrated using the continuous phase transition extraction device and mixed with anhydrous ethanol (1:4, v/v) at 4^°^C overnight. The precipitation was collected by centrifugation at 2,620 G for 10 min and freeze-dried to obtain the crude GLP.

The GLP was dissolved in 500 ml of distilled water and centrifuged at 2,620 G for 10 min before loading on a 10 kDa cut-off ultrafiltration membrane. GLP solution sample was injected into the cut-off ultrafiltration membrane at a rate of 1 ml/min by a constant current pump for ultrafiltration separation. In this way, an eluted fraction (EGLP, Mw < 100 kDa) and a retained (RGLP, Mw > 100 kDa) fraction were obtained according to molecular weight. Then EGLP and RGLP fractions were lyophilized and stored at −20^°^C.

The RGLP was further purified by Sephacryl S-500 HR column (2.6 × 60 cm), pre-equilibrated with deionized water, and eluted with 0.2 mol/L NaCl at a rate of 0.8 ml/min (8 ml/tube). The main fraction was collected, dialyzed, and lyophilized to afford one white fluffy polysaccharide RGLP-1. The polysaccharide content was determined by the phenol-sulfuric acid method.

### Study on the Immunostimulatory Activity of Polysaccharides

#### Culture Cell

RAW264.7 cell lines were cultured in DMEM supplemented with 10% heat-inactivated FBS containing 100 μg/ml of penicillin and 100 μg/ml of streptomycin at 37°C under humidified air with 5% CO_2_. Cells in the exponential growth phase were used for experiments.

#### Cell Viability Analysis

The effect of polysaccharide GLP, EGLP, and RGLP on the viability of RAW264.7 cells was determined by MTT assay. The cells were seeded at a density of 1 × 10^5^ cells/ml in a 96-well plate for 24 h at 37°C in a humidified atmosphere with 5% CO_2_, and then were cultured with different concentrations of polysaccharides (1, 2, 5, 10 μg/ml) for 24 h. Each concentration was repeated in three wells. After incubation, MTT was added to each well 20 μl to a final concentration of 0.5 mg/ml. After 4 h incubation, the produced formazan crystals were dissolved in DMSO (150 μl/well). The absorbance was measured at 570 nm, and the cell proliferation index was calculated according to the formula:

Cell proliferation index (%) = (*A*_1_/*A*_0_) × 100%. *A*_0_ is the absorbance of a blank treatment group. *A*_1_ is the absorbance of a sample treatment group.

#### Phagocytic Activity Assay

The phagocytic activity of GLP, EGLP, and RGLP in RAW264.7 cells was measured by a neutral red assay. RAW264.7 cells were seeded at 2 × 10^4^ cells/well in a 96-well plate and incubated for 24 h. Various final concentration of GLP, RGLP, and EGLP (1, 2, 5, 10 μg/ml), DMEM medium and LPS (1 μg/ml) were added into each well. After 24 h incubation in the condition of 37°C and 5% CO_2_, culture media were removed and 100 μl/well of 0.1% neutral red was added and incubated for 4 h, the plate was washed with PBS for three times, 100 μl of cell lying solution (ethanol and acetic acid mixed in equal volume) was added into each well and shaken for 10 min. The absorbance was evaluated in a microplate reader at 540 nm, and the phagocyte phagocytosis rate was calculated according to the equation:

Phagocyte phagocytosis index (%) = (*A*_1_/*A*_0_) × 100%. *A*_0_ is the absorbance of a blank treatment group. *A*_1_ is the absorbance of a sample treatment group.

#### Determination of Nitric Oxide, Tumor Necrosis Factor-α, and Interleukin-6

The RAW264.7 cells were seeded into 96-well plates at a density of 2 × 10^4^ cells/well and cultured for 24 h with GLP, EGLP, and RGLP at various concentrations as mentioned earlier. Equal volume of medium or LPS (1 μg/ml) was added as the control and positive group, respectively. Then, the cell culture supernatants were collected and levels of nitric oxide (NO), tumor necrosis factor-α (TNF-α), and interleukin 6 (IL-6) were measured by the Nitric Oxide Assay kit (Beyotime, Shanghai, China) and TNF-α, IL-6 enzyme-linked immunoassay kits (Neobioscience, Shenzhen, China).

### Characterization of RGLP-1

#### Ultraviolet Absorption Spectroscopy Analysis

To detect the purity of the RGLP-1, the UV spectra of RGLP-1 were investigated using an UV spectrophotometer (UV-1700, Shimadzu, Japan) scanned in the 200–400 nm region according to a published method with minor modification (23). Briefly, 1.0 mg/ml RGLP-1 was prepared with distilled water, the solution was transferred in a quartz cuvette.

#### Determination of Molecular Weight

The molecular weight of RGLP-1 was measured using HPGPC (24) performed on an analytic HPLC system (Shimadzu) equipped with a TSK G-6000 PWXL column (7.8 × 300 mm) connected to TSK G-3000 PWXL column (7.8 × 300 mm, Tosoh Biosep, Japan) and a RID-10A refractive index detector (Shimadzu). The injection volume was 20 μl. The 0.02 mol/L Na_2_SO_4_ solution was used as mobile phase at a flow rate of 0.6 ml/min at 35°C. The average molecular weight of RGLP-1 was calculated by the calibration curve established by a series of dextran standards (5, 25, 40, 80, 270, 410, 670, 1,500, and 1,800 kDa).

#### Monosaccharide Composition

To determine the monosaccharide composition, RGLP-1 was first hydrolyzed using a reported procedure as previously described method (25). The RGLP-1 was hydrolyzed by 4 ml of 2 mol/L trifluoroacetic acid (TFA) at 110^°^C for 6 h. Acetylation was carried out with 10 mg of hydroxylamine hydrochloride and 1 ml of pyridine for 30 min at 90^°^C. Next, 1 ml of acetic anhydride was added with continuous heating. The hydrolysis products were converted into their acetylated derivatives and analyzed by GC-MS spectrometer (TSQ 8000, Thermo Fisher, Waltham, MA, United States) equipped with a TG-5MS capillary column (60 m × 0.25 mm × 0.50 μm). Five monosaccharides (fucose, mannose, rhamnose, galactose, and glucose) were used as the external standards to identify the composition of the polysaccharides.

The temperature program of the column was set as follows: initial temperature of 160^°^C for 2 min, then increased to 200^°^C at the rate of 5°C/min and held for 5 min. Subsequently, the temperature increased from 200 to 230^°^C at 2^°^C/min and was maintained for 10 min. Helium was used as the carrier gas at a constant flow rate of 0.8 ml/min. The injection temperature and detector temperature were both 250^°^C. The injection volume was 1 μl and the split ratio was 10: 1 (26).

#### Periodate Oxidation—Smith Degradation

The samples were treated using a modified method (27). Briefly, the RGLP-1 (10 mg) was dissolved in 12.5 ml of distilled water, and 12.5 ml of sodium periodate (30 mmol/L) was then added. The solution was kept in the dark at 4°C, and 0.1 ml of aliquots were withdrawn at 6–12 h intervals, diluted to 25 ml with distilled water, and read using a spectrophotometer at 223 nm, until the optical density value became stable. Glycol (2 ml) was used to stop periodate oxidation. The solution of periodate product (2 ml) was titrated to calculate the production of formic acid by 0.01 mol/L sodium hydroxide, and the rest was extensively dialyzed against tap water and distilled water for 48 h. The residues were concentrated and reduced with sodium borohydride (35 mg). The solution was placed at room temperature for 24 h, neutralized to pH 5.5 with 50% acetic acid, dialyzed as described earlier, and concentrated to a volume of 10 ml followed by freeze-drying. Subsequent treatments were performed as described previously. The residues (10 mg) were hydrolyzed with trifluorocetic acid and then were acetylated with hydroxylamine hydrochloride, pyridine, and acetic anhydride.

#### Methylation Analysis

The polysaccharide was methylated by a published method with some modification (28). The dried RGLP-1 (10 mg) was dissolved in 5 ml DMSO with heated or sonicated for 1 h to ensure a completed solution. Sodium hydroxide (200 mg) and iodine methane (2.5 ml) were added to the solution with stirring for 12 h in a dark place. This methylation procedure was conducted three times and stopped by the addition of 2 ml distilled water. The methylated polysaccharide was extracted three times with 5 ml trichloromethane. The trichloromethane extract was then evaporated to dryness. The dried methylated polysaccharide was hydrolyzed with trifluorocetic as described earlier. The hydrolysate was dissolved in 4 ml distilled water and the pH value in solution should be adjusted to 10–12 by 10% sodium hydroxide solution. Then, the hydrolysate solution was reduced by sodium borohydride (100 mg). After 12 h, the pH value in solution should be adjusted to 5.5 by 50% acetic acid and evaporated to dryness. The following procedure of acetylation was described as earlier.

#### Fourier Transform Infrared Spectra Analysis

The infrared profile of RGLP-1 was determined using the KBr disc method. The KBr-disk was monitored in the range of 400–4,000 cm^–1^ with a Fourier transform infrared (FT-IR) spectrophotometer (Vertex70, Bruker, Ettlingen, Germany). The experimental parameters setting of resolution was 4 cm^–1^ and the scans number was 16 times.

#### Scanning Electron Microscope Observation

Morphological alterations of RGLP-1 were observed using an EVO18 scanning electron microscope (ZEISS, Germany) at an accelerating voltage of 10.0 kV. The dried sample was coated with a gold film before testing, and scanned at the magnification of 250×, 500×, 1,000×, and 2,000×.

#### Atomic Force Microscopy Observation

The dried sample was dissolved to 10 μg/ml deionized water. About 10 μl of polysaccharides solution was dropped onto a mica plate and dried at room temperature for 12 h. The AFM (Multimode 8 instrument, United States) was operated in the tapping mode. The images were captured by NanoScope software.

#### Congo Red Test

The helical structure of RGLP-1 was determined *via* Congo red test according to a reported method with minor modification (29). In brief, 2.0 ml polysaccharide sample (1 mg/ml) was mixed with 2.0 ml of a Congo red solution (80 μmol/L). Then, the mixtures were gradually added with a 1 mol/L NaOH solution to form the final NaOH concentrations of 0–0.5 mol/L. The mixtures were equilibrated for 10 min at room temperature. The maximum absorption wavelength of Congo red and Congo red-polysaccharide mixture was measured by an ultraviolet-visible spectrophotometer (UV-1700, Shimadzu, Japan) in the range of 400–600 nm.

#### Circular Dichroism Analysis

The CD spectra of RGLP-1 (1 ml of 1 mg/ml polysaccharide solution was mixed with 1 ml deionized water) and RGLP-1-Congo red complex (1 ml of 1 mg/ml polysaccharide solution was mixed with 1 ml of 80 μmol/L Congo red) were obtained on a chirascan CD spectrometer (Applied Photophysics Ltd., United Kingdom) with a scan rate of 100 nm/min and a bandwidth of 1 nm and a time constant of 1 s at 25^°^C. Data were collected from 190 to 260 nm with three scans averaged.

### Statistical Analysis

All experimental data were expressed as the mean ± *SD*. The significance of the difference was evaluated by multiple comparison analyses with Duncan’s tests (SPSS 21.0 software). A value of *P* < 0.05 was considered to be defined as statistically significant.

## Results and Discussion

### Extraction, Purification, and Molecular Weight Distribution of *Ganoderma lucidum* Polysaccharides

**FIGURE 1:**
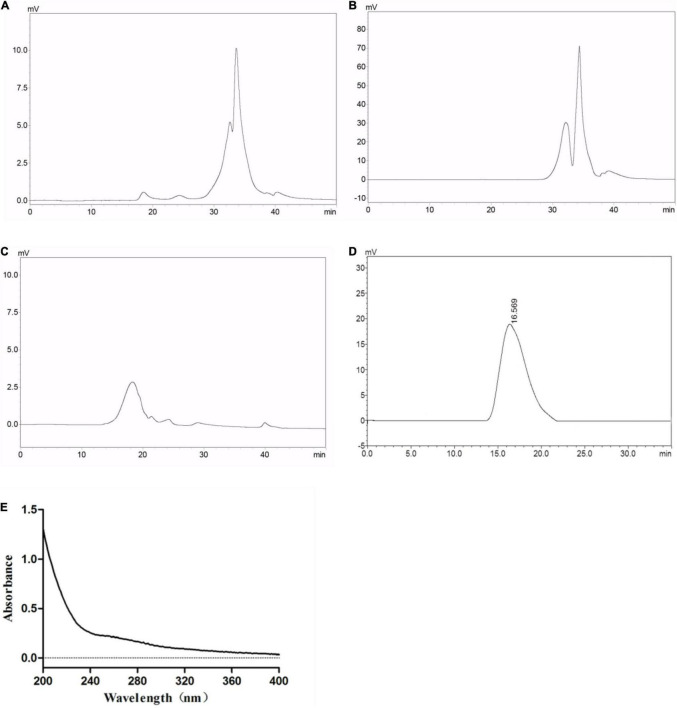
Average molecular weight distribution of **(A)** GLP, **(B)** EGLP, **(C)** RGLP, **(D)** RGLP-1 and ultraviolet absorption spectrum **(E)** of RGLP-1.

Crude GLP with a yield of 7.13% was isolated from *G. lucidum* through hot water extraction using continuous phase transition extraction technology followed by ethanol precipitation and lyophilization. The molecular weight distribution of the crude GLP was determined by HPGPC as illustrated in Figure 1A. HPGPC chromatograms profiles of GLP exhibited two main populations at the retention time of 32 min and 34 min. The percentage of the two populations with low molecular weight was 95.26%, implying that GLP mainly contained polysaccharides with low molecular weight. In crude GLP, two small populations at the retention time of 18.5 and 24 min were also found which only accounted for 4.74%. GLP was then purified by a 10 kDa cut-off ultrafiltration membrane into two fractions, including EGLP (Figure 1B) and RGLP (Figure 1C). According to the calibration curve (log Mw = 12.671–0.292t, *R*^2^ = 0.9989). EGLP consisted of two main fractions with an average molecular weight of 3,288 and 948 Da. In the RGLP fraction, one main population at 3,473 kDa was obtained representing 95% according to the area normalization method in Figure 1C. A previous study reported that the crude polysaccharides had three major fractions (PS-F1, PS-F2, and PS-F3), PS-F1 contained large polysaccharides which are over 2,000 kDa, and PS-F2 (6–52 kDa) and PS-F3 (2–4 kDa) contained smaller molecule polysaccharide (30). Different molecular weight distributions may be due to the difference between the extraction process and the alcohol precipitation process.

Restriction Fragment Length Polymorphism with higher immunomodulatory activity was further purified through the Sephacryl S-500 HR column. A novel purified fraction of RGLP-1 was obtained and the molecular weight of RGLP-1 was determined by HPGPC in Figure 1D. It could be seen that RGLP-1 exhibited a narrow and symmetrical peak, of which the baseline was stable, indicating that RGLP-1 was a homogeneous polysaccharide after purification by cut-off ultrafiltration membrane and Sephacryl gel column. According to the calibration curve, the molecular weight of RGLP-1 was exactly 3,978 kDa. RGLP-1 contained 96.23% (w/w) of total sugar by the phenol–sulfuric acid method. UV absorption spectrum of RGLP-1 showed no absorption peak at 260 or 280 nm in Figure 1E, indicating that RGLP-1 was absent of nucleic acids and protein.

### *In vitro* Immunostimulatory Activities

#### Effects of *Ganoderma lucidum* Polysaccharides, EGLP, and RGLP on the Proliferation of Macrophages

In this study, the RAW264.7 macrophage cells model was used to perform the immunomodulatory activity of GLP, EGLP, and RGLP. For each sample, four concentrations (1, 2, 5, and 10 μg/ml) were selected.

**FIGURE 2:**
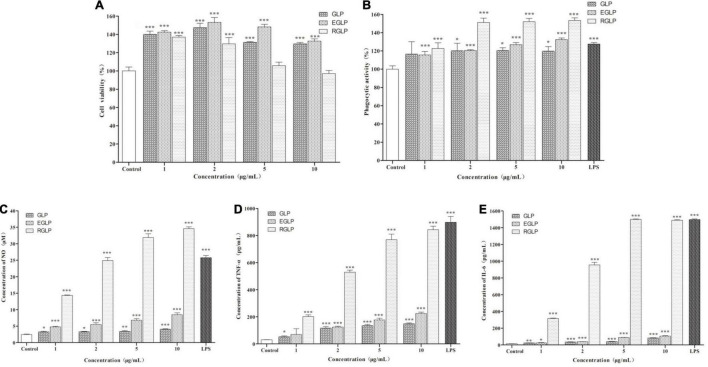
The effect of polysaccharides on cell viability **(A)**, phagocytic ability **(B)**, NO **(C)**, TNF-α **(D)**, and IL-6 **(E)** of RAW 264.7 cells. ***Statistically significant at *P* < 0.001. **Statistically significant at *P* < 0.01. *Statistically significant at *P* < 0.05.

The MTT assay indicated that GLP, EGLP, and RGLP had no cytotoxic effect on macrophage RAW264.7 cells at a concentration range of 1–10 μg/ml in Figure 2A. At the concentration of 2 μg/ml, EGLP had the best proliferative effect of RAW264.7 cells. RGLP had the best proliferation on RAW264.7 cells in the concentration of 1 μg/ml and the viability showed a slight decline when the concentrations increased further from 1 to 10 μg/ml. Therefore, the selected concentration of GLP, EGLP, and RGLP for immunological activity assay should fall below 10 μg/ml.

#### Effect of *Ganoderma lucidum* Polysaccharides, EGLP, and RGLP on the Phagocytosis of Macrophages

Phagocytosis is one of the most prominent characteristics of macrophage activation and is an important barrier to the host innate immune system (31). As shown in Figure 2B, LPS (1 μg/ml) as the positive control could significantly promote RAW264.7 cells to uptake neutral red significantly (*p* < 0.001). As expected, compared with the control, GLP, EGLP, and RGLP also enhanced the phagocytic ability significantly at the concentration range of 1–10 μg/ml. The extent of stimulation followed the order: RGLP > EGLP > GLP. The phagocytic ability in RGLP at the concentration range of 2–10 μg/ml was significantly higher than that of LPS (*p* < 0.01).

#### Effects of *Ganoderma lucidum* Polysaccharides, EGLP, and RGLP on the Production of NO and Cytokines From RAW264.7 Macrophages

Activated macrophages secrete NO and cytokines, such as TNF-α and IL-6, which play an important role in immunomodulation (32). The results showed that compared with the control group, GLP, EGLP, and RGLP could significantly stimulate the NO production in a dose-dependent manner (*p* < 0.001) in Figure 2C. NO production in RGLP was higher than those of GLP and EGLP groups.

Besides, the expressions of TNF-α and IL-6 were in a dose-dependent manner with the increase of the GLP, EGLP, and RGLP concentration in Figures 2D,E. When the concentration of GLP, EGLP, and RGLP was 10 μg/ml, the relative expression content of TNF-α reached the maximal, respectively, was 4.85, 7.66, and 27.50 times higher than that of the control group. Like the result of TNF-α, the relative expression content of IL-6 of GLP, EGLP, and RGLP also reached the maximal at the concentration of 10 μg/ml, which was 5.55, 6.98, and 98.56 times higher than that of the control group. The amount of NO, TNF-α, and IL-6 stimulated by each fraction followed the order: RGLP > EGLP > GLP. Overall, these results suggested that GLP, EGLP, and RGLP may potentially enhance macrophage-mediated immune response, especially RGLP.

### Structural Analysis

#### Monosaccharide Composition

**FIGURE 3:**
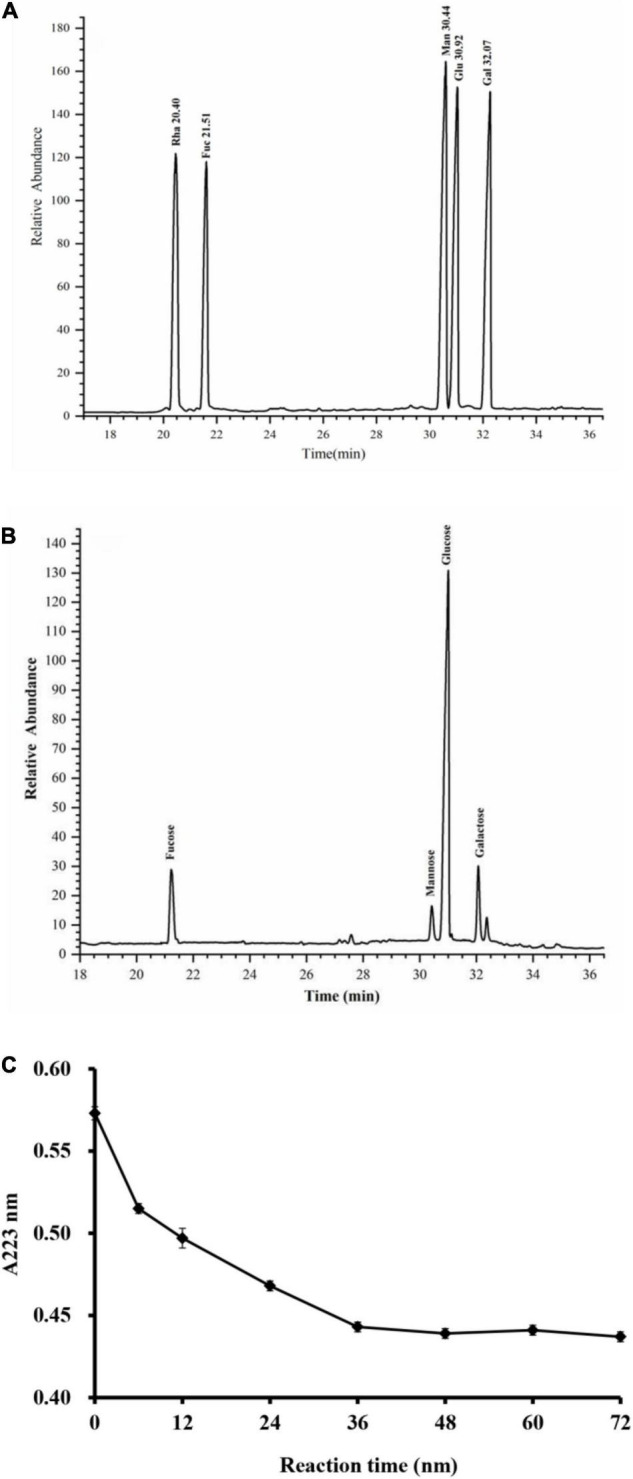
GC–MS chromatograms of monosaccharide standards **(A)** and RGLP-1 **(B)**; The periodic acid consumption of RGLP-1 **(C)**.

As shown in Figure 3, the monosaccharide composition of RGLP-1 was composed of fucose, mannose, glucose, and galactose at a molar ratio of 0.13: 0.05: 0.72: 0.10. These results suggested that RGLP-1 is a heteropolysaccharide, glucose had the highest proportion and may be the backbone of the chain of RGLP-1. A previous study found that GLP-3 was purified from *Ganoderma leucocontextum* by ultrafiltration and column chromatography consisted of arabinose, xylose, mannose, glucose, and galactose in a molar ratio of 2.4: 3.3: 0.8: 92.7: 0.8 (33). In addition, the purified GLP consist of glucose, galactose, arabinose, xylose, and mannose with molar ratios of 0.793: 0.964: 2.944: 0.167: 0.384: 7.94 and linked by β-glycosidic linkages (34). Although the heterogeneous polysaccharides were obtained after separation from *G. lucidum*, there were significant differences in the types and amount of monosaccharides. This is likely because of the differences in varieties of raw materials, extraction process, and purification method.

#### Periodate Oxidation and Smith Degradation Analysis

Periodate oxidation results are shown in Figure 3C. About 1 mol of hexose residue consumed 0.358 mol of periodate and produced 0.016 mol of formic acid. A small amount of formic acid formation suggested that RGLP-1 contains a small amount of (1→) or (1→6) linkage. The molar amount of periodate consumed by RGLP-1 was far less than the sample consumption, it indicated that there is a large proportion of (1→3) linkage in RGLP-1. The molar amount of periodate consumed by RGLP-1 was far more than two times the molar amount of formic acid, revealing that RGLP-1 also contains (1→2) or (1→4) linkage.

Then RGLP-1 structure was further analyzed by Smith degradation by GC–MS. The glycerol, erythritol, mannose, glucose, and galactose absorption peak were found. Glycerol indicated that (1→2), (1→4), or (1→6) linkage existed. Erythritol is a chemical indicative of (1→4) or (1→6) linkage, suggesting that (1→4) or (1→6) linkage existed. The presence of a lot of mannose, glucose, and galactose demonstrated that some of the linkages, such as (1→3), (1→3,6), (1→2,3), (1→2,4), (1→3,4), and (1→2,3,4), were existed (35–37). This finding is consistent with a previous study that GCPB-2 (GLP fraction) may have (1→2), (1→4), (1→6) glycosidic bonds by periodate oxidation and Smith degradation analysis (37). More precise glycosidic bonds were confirmed in the methylation analysis.

#### Methylation Analysis

The methylation analysis was performed to determine the glycosidic linkages of RGLP-1. Based on the retention time and standard data in the CCRC spectral database, RGLP-1 exhibited 12 main peaks as summarized in Table 1.

**TABLE 1:** GC–MS analysis of the methylated products of RGLP-1.

Retention time (min)	Methylated sugar	Linkage	Molar ratio
20.31	2,3,4-Me_3_-Galp	1,6-	1.50
21.12	2,4,6-Me_3_-Manp	1,3-	1.41
21.64	2,3,4,6-Me_4_-Glcp	T-	22.40
22.19	2,3,4,6-Me_4_-Galp	T-	2.12
24.39	3,4,6-Me_3_-Manp	1,2-	0.56
24.57	2,4,6-Me_3_-Glcp	1,3-	34.54
24.87	2,3,6-Me_3_-Glcp	1,4-	8.11
25.16	2,4,6-Me_3_-Galp	1,3-	4.13
25.64	2,3,4-Me_3_-Glcp	1,6-	4.12
27.72	4,6-Me_2_-Galp	1,2,3-	2.16
29.12	2,3-Me_2_-Galp	1,4,6-	1.14
29.45	2,4-Me_2_-Glcp	1,3,6-	17.81

According to the retention time, the 12 peaks were identified as 2,4,6-Me_3_-Glcp, 2,3,4,6-Me_4_-Glcp, 2,4-Me_2_-Glcp, 2,3,6-Me_3_-Glcp, 2,3,4-Me_3_-Glcp, 2,3,4-Me_3_-Glcp, 2,4,6-Me_3_-Galp, 4,6-Me_2_-Galp, 2,3,4,6-Me_4_-Galp, 2,3,4-Me_3_-Galp, 2,3-Me_2_-Galp, 2,4,6-Me_3_-Manp, and 3,4,6-Me_3_-Manp with a molar ratio of 34.54:22.40:17.81:8.11:4.12:4.13:2.16:2.12:1.50:1.14:1.41:0.56. The results suggested that RGLP-1 contained 12 linkage forms: (1→3)-linked glucose, (1→)-linked glucose, (1→3,6)-linked glucose, (1→4)-linked glucose, (1→6)-linked glucose, (1→3)-linked galactose, (1→2,3)-linked galactose, (1→)-linked galactose, (1→6)-linked galactose, (1→4,6)-linked galactose, (1→3)-linked mannose, and (1→2)-linked mannose. These results indicated that (1→3)-linked glucose, (1→3,6)-linked glucose, and (1→4)-linked glucose was the main chain of RGLP-1 with a small amount of (1→6) linkage. The above results also agreed with the result from the periodate oxidation–Smith degradation. This result was consistent with a previous study that the main chain of water-soluble β-glucan from *G. lucidum* is 1, 3-linked Glcp (38).

The presence of terminal residues (T-Glcp and T-Galp) and branching points [(1→3, 6)-linked-Glcp and (1→4, 6)-linked-Galp] indicated that RGLP-1 contained both linear and branched polysaccharides. The ratio between terminal residues and the branching points was 1.16, suggesting that the number of terminal units was slightly more than that of branching points. Moreover, the degree of branching (DB) value of RGLP-1 was 45.63% calculated by the equation DB = (NT + NB)/(NT + NB + NL), where NT is the numbers of the terminal residues (T-Glcp and T-Galp), where NB is the numbers of the branch residues [(1→3, 6)-linked-Glcp and (1→4, 6)-linked-Galp], and where NL is the linear residues (1,3-Glcp, 1,4-Glcp, 1,6-Glcp, 1,3-Galp, 1,3-Galp, 1,6-Galp).

#### Fourier Transform-Infrared Spectroscopy Analysis

**FIGURE 4:**
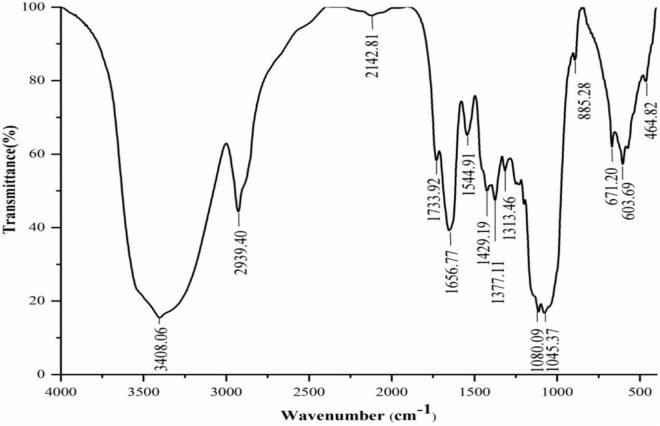
IR spectra of RGLP-1.

The FT-IR spectrum of RGLP-1 exhibited typical signals for polysaccharides in the range 4,000–400 cm^–1^, and the characteristic absorption peak was apparent in Figure 4. A strong band at 3,408.06 cm^–1^ was the –OH group. The band at 2,939.40 cm^–1^ was due to the C–H stretching vibration (39). The absorbance at 1,656.77 cm^–1^ indicated the presence of the bound water (40). The four peaks at 1,544.91, 1,429.19, 1,377.11 and 1,313.46 cm^–1^ can be assigned to methyl C–H wagging vibrations (41). The region around 1,045–1,080 cm^–1^ indicated the presence of a pyranose ring structure. Moreover, the band at 885.28 cm^–1^ is the characteristic absorption of β-linkage pyranose (37). The spectra indicated that RGLP-1 had the typical groups of sugars.

#### Scanning Electron Microscopy Observation

**FIGURE 5:**
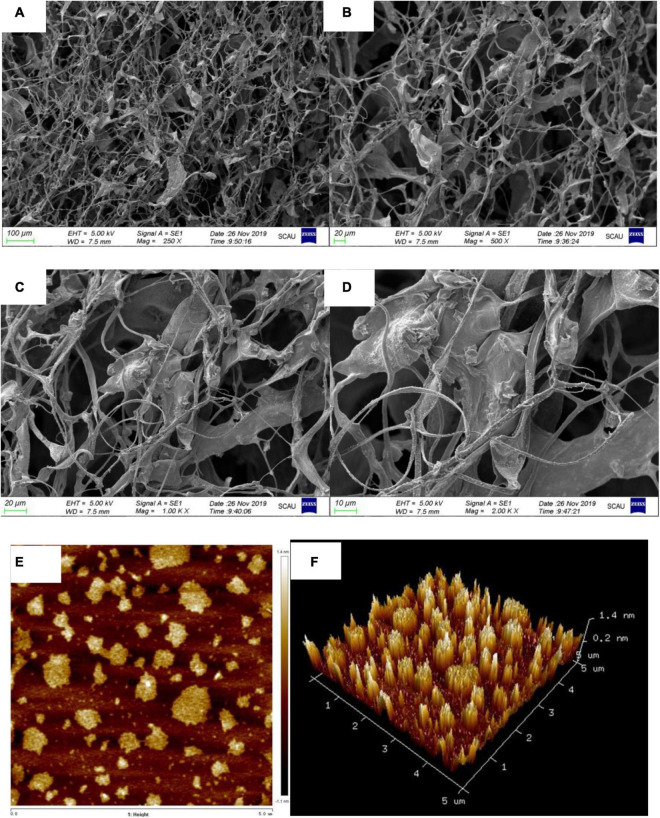
SEM image with different magnifications ×250 **(A)**; ×500 **(B)**; ×1,000 **(C)**; ×2,000 **(D)**; AFM height analysis images **(E)**; and three dimensional image **(F)** of RGLP-1.

Restriction Fragment Length Polymorphism-1 units formed filiform-shaped chains with uneven diameters at the ends, generating a fibrous structure with characteristic interconnection or aggregates in Figures 5A–D. When this specific filiform-shaped surface was enlarged to × 2,000 times, the surface was smooth and dense with many spiral folds. This finding is similar to the morphology analysis of purified *Dendrobium aphyllum* polysaccharide (42). It indicated that RGLP-1 with higher molecular weight showed a strong intermolecular interaction and existed crosslinking which might be related to its complex structure.

#### Atomic Force Microscopy Analysis

Atomic force microscopy is a powerful technique to characterize soft biological surfaces, has gradually been used to analyze the surface of biological macromolecules (43, 44). The AFM diagrams of RGLP-1 are presented in Figures 5E,F, respectively. It is clear that the polysaccharide displayed irregular globular-like or curly structure. The diameters of the molecules were ranging from 110.2 to 706.9 nm and the heights were in the range of 0.9–6.0 nm. Normally, the height of a single polysaccharide chain is 0.1–1 nm. We speculate that the branched molecular chains of RGLP-1 were intertwined with each other to form circular aggregates. The hydroxyl groups in the RGLP-1 molecule may be the most important factor for the strong intramolecular and intermolecular interaction to form aggregates on the mica sheet (45).

#### Congo Red Test

**FIGURE 6:**
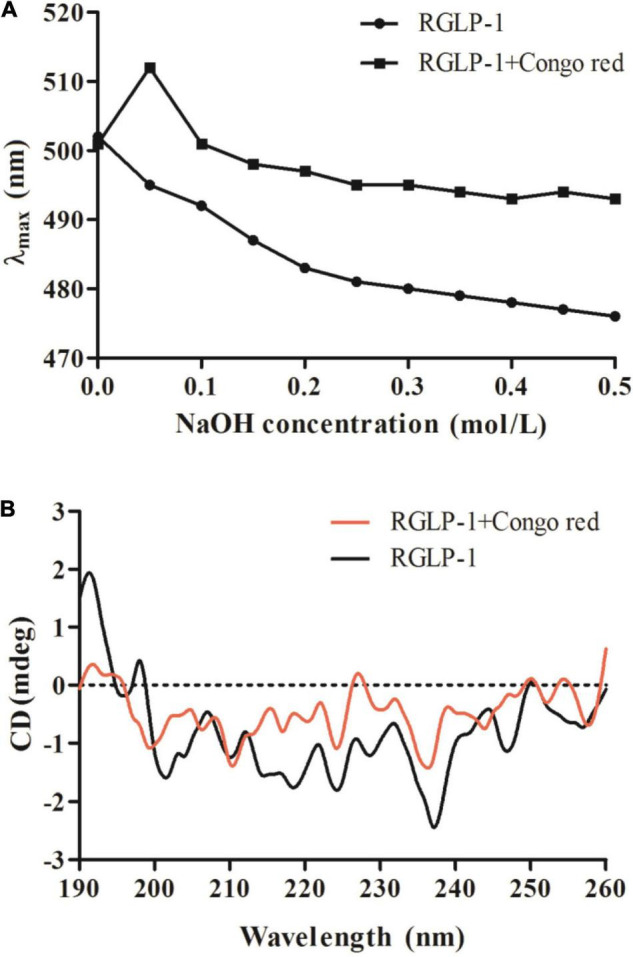
Maximum absorption wavelength of RGLP-1 and RGLP-1 + Congo red at different concentrations of NaOH **(A)**; CD spectrogram **(B)** of RGLP-1.

Generally, triple-helix polysaccharides combined with Congo red will lead to the redshift of the maximum absorption wavelength (λ_max_) of the Congo red-polysaccharide complex. Therefore, the existence of triple-helix structure of polysaccharides can be determined by Congo-red experiment. It was reported that original blackberry fruiting polysaccharide had triple-helical conformation using the Congo-red method (46). It was apparently observed that the interaction of Congo red-RGLP-1 polysaccharide complex undergo a distinct bathochromic shift from 501 to 512 nm in 0.05 mol/L NaOH solution in Figure 6A, the maximum absorption wavelength decreased with the increase of NaOH concentration, which indicated that RGLP-1 had a triple-helix structure. The recent study also reported that triple-helix structure existed in GLP-3 purified from *G. leucocontextum* (33).

#### Circular Dichroism Analysis

To further investigate the conformational structure information of RGLP-1, the CD spectroscopy was obtained in the range of 190–260 nm. As shown in Figure 6B. RGLP-1 had a positive cotton effect at 192 and 197 nm indicating that the polysaccharide was ordered. In addition, the negative cotton peak at 202 nm in the spectra indicating that RGLP-1 was existed by ordered helical structure in water solution. When Congo red was presented, the signals in the CD spectra had shifted. In comparison with the maximum peak of RGLP-1, the positive cotton effect of the Congo red-polysaccharide complex weakened a lot and the negative cotton effect disappeared. This result further proved that RGLP-1 has a triple-helical structure, which was consistent with the Congo red experimental analysis.

## Conclusion

In summary, we screened a homogeneous and high molecular weight polysaccharide (RGLP-1) with the greatest immunoregulatory activity *in vitro* after double purification with ultrafiltration membrane and Sephacryl S-500 HR. RGLP-1 had a relative molecular weight of 3,978 kDa. RGLP-1 was composed of fucose, mannose, glucose, and galactose at a molar ratio of 0.13: 0.05: 0.72: 0.10, the majority of which was (1→3)-β-Glcp and (1→3, 6)-β-Glcp residues with triple-helix conformation. RGLP-1 showed a strong intermolecular interaction and diameters of the molecules was ranging from 110.2 to 706.9 nm and heights in the range of 0.9–6.0 nm. Thus, these findings explored the structure-activity relationship of GLP and suggested that RGLP-1 could be a complementary dietary bioactive component for immunoregulation.

## Data Availability Statement

The original contributions presented in the study are included in the article/supplementary material, further inquiries can be directed to the corresponding author/s.

## Author Contributions

GL: data curation, formal analysis, investigation, writing—review, and editing, validation. JZ: data curation, formal analysis, investigation, and roles and writing—original draft. QK: data curation, formal analysis, writing—review, and editing. MS: data curation, formal analysis, and investigation. TH: data curation, formal analysis, and investigation. SA: data curation and formal analysis. HL: investigation and methodology. HC and LH: formal analysis, methodology, and resources. JX: writing—review and editing. YJC: investigation, writing—review and editing, and supervision. YNC: project administration, funding acquisition, writing—reviewing and editing, investigation, supervision, and resources. All authors contributed to the article and approved the submitted version.

## Conflict of Interest

HC and LH were employed by Infinitus China Co., Ltd. The remaining authors declare that the research was conducted in the absence of any commercial or financial relationships that could be construed as a potential conflict of interest.

## Publisher’s Note

All claims expressed in this article are solely those of the authors and do not necessarily represent those of their affiliated organizations, or those of the publisher, the editors and the reviewers. Any product that may be evaluated in this article, or claim that may be made by its manufacturer, is not guaranteed or endorsed by the publisher.
